# Immunohistochemical Detection of CD147 Expression in Adenocarcinoma of the Prostate: A Case-Control Study

**DOI:** 10.1155/proc/4406057

**Published:** 2024-12-22

**Authors:** Sara Suliman, Mona Ellaithi

**Affiliations:** Department of Histopathology and Cytology, Faculty of Medical Laboratory Sciences, Al-Neelain University, Khartoum, Sudan

## Abstract

Prostate cancer is the most common noncutaneous malignancy among men worldwide, including in Sudan, where it represents a significant public health challenge. CD147, a transmembrane glycoprotein implicated in tumor progression, invasion, and metastasis, has shown potential as a prognostic biomarker in various cancers. This retrospective case-control study aimed to evaluate CD147 expression in prostate adenocarcinoma among Sudanese men and its association with tumor grade. A total of 80 paraffin-embedded tissue samples, including 40 cases of prostate adenocarcinoma and 40 benign prostatic hyperplasia (BPH) controls, were analyzed using immunohistochemistry. CD147 expression was observed in 22.5% of adenocarcinoma cases compared to 7% of controls; however, the association was not statistically significant (*p*=0.07). Low-grade tumors were predominant in the cohort, consistent with early-stage diagnoses. The findings revealed no clear link between CD147 expression and tumor grade, diverging from prior studies that associate CD147 with advanced tumor stages. The nonsignificant results may be attributed to the small sample size, emphasizing the need for future research with larger, more diverse cohorts, advanced molecular techniques, and functional studies to better elucidate the role of CD147 in prostate cancer pathogenesis and its potential as a therapeutic target.

## 1. Introduction

Prostate cancer is the most common noncutaneous malignancy among males worldwide, with approximately 1.6 million new cases and 366,000 deaths annually [1]. According to more recent data from GLOBOCAN 2020, prostate cancer remains a leading cause of cancer-related morbidity and mortality globally, particularly in regions such as Australia/New Zealand, Northern America, and Western and Northern Europe. The incidence is also notably high in less developed regions such as the Caribbean, Southern Africa, and South America, while rates remain lower in Asian populations [2].

Prostate cancer is the most prevalent cancer among Sudanese men [3], with an age-standardized incidence rate of 10.3 per 100,000 and a mortality rate of 8.7 per 100,000 population. It ranked second among all cancers in both sexes, following breast cancer, in 2012 [4], prostate cancer was much less common, ranking 10th among cancers diagnosed in men at the Sudan Cancer Registry in 1978, trailing behind skin cancers and non-Hodgkin lymphoma [5]. At that time, it accounted for only 0.8% of all male cancers (out of 10,410 cases) studied at RICK between 1967 and 1984 [6]. By 2000–2006, prostate cancer had become the most commonly diagnosed cancer in men, comprising 7.6% of all male cancer cases (out of 10,911 cases) at RICK and NCI-UG [7]. More recently, prostate cancer has AE leading cancer among male patients treated at NCI-UG [8], ranking first among cancers in men (*n* = 268) treated at the NCI in central Sudan from 2006 to 2009. Most cases (85.4%) were diagnosed at advanced stages (Stage III or IV), with a mean patient age of 72.2 ± 9.25 years [9]. The current increase in cases might be attributed to advancements in diagnostic techniques now available in the country.

In Sudan, prostate cancer is the most frequently diagnosed malignancy among men, affecting individuals across various ethnic groups. Most Sudanese men present with advanced-stage disease (Stage III or IV) at a mean age of 72.2 years, making early detection and treatment challenging [3].

Cluster of differentiation 147 (CD147), also known as extracellular matrix metalloproteinase inducer (EMMPRIN) or basigin (BSG), is a transmembrane glycoprotein encoded by the BSG gene [10–12]. As a member of the immunoglobulin superfamily [12, 13], CD147 plays a significant role in intercellular recognition and signaling [14]. CD147 functions as a Type I integral membrane receptor and interacts with several ligands, including cyclophilin proteins, *Plasmodium falciparum* reticulocyte binding-like homolog 5 (PfRh5), and integrins [15].

CD147 is extensively expressed in various human malignancies and is implicated in cancer progression by promoting the release of MMPs and cytokines. It influences critical processes such as cell proliferation, apoptosis, tumor cell migration, metastasis, and differentiation, particularly under hypoxic conditions [15]. CD147 is among the most commonly overexpressed proteins in metastatic cancer cells and has been linked to a poor prognosis in several types of cancer. Overexpression of CD147 has been observed in malignancies such as glioma [16, 17], breast cancer [18, 19], cervical cancer [20], colon cancer [21], and endometrial cancer [22]. Recent studies have also highlighted CD147's role in facilitating tumor immune evasion and enhancing tumor aggressiveness, making it a potential target for therapeutic intervention [23, 24].

In prostate cancer, previous studies have reported increased CD147 expression in tumor samples compared to matched benign tissues, with an association to a poorer prognosis following prostatectomy [25–27]. However, two recent studies have shown conflicting results, with a decrease in CD147 expression observed as prostate cancer progresses [27, 28], suggesting a potential protective role for CD147 in this context. The prognostic significance of CD147 in prostate cancer remains controversial, with inconsistent findings across studies [27–30]. In addition, the sensitivity of previous investigations has been limited by the semiquantitative nature of immunohistochemical analysis methods. The aim of this study is to investigate the expression of CD147 and its potential role as a independent biomarker for prostate cancer.

## 2. Materials and Methods

### 2.1. Samples

This retrospective case-control study aimed to analyze CD147 expression in Sudanese men diagnosed with prostate adenocarcinoma. A total of 80 paraffin-embedded tissue samples were included, comprising 40 samples from cases with different grades of prostate adenocarcinoma and 40 samples from patients with benign prostatic hyperplasia (BPH).

### 2.2. Ethical Consideration

The study protocol was reviewed and approved by the Central Institutional Review Board of Al-Neelain University (IRB serial number: NU-IRB-17-581). All research procedures followed ethical guidelines to protect participants' privacy and confidentiality.

### 2.3. Methods

Sections of 3-4 micron thickness were prepared from the paraffin blocks. The paraffin was removed, and the sections were placed in an antigen retrieval buffer. The sections were then incubated in a water bath before being transferred to a preheated buffer for 30 min, followed by cooling at room temperature for 15 min. After washing in water, the sections underwent antigen retrieval by steaming in a high pH (9) buffer at 95°C for 40 min. Following a brief wash with phosphate-buffered saline (PBS), endogenous peroxidase activity was blocked using 3% hydrogen peroxide in methanol for 10 min.

After another PBS wash, the sections were incubated with 100 μL of a mouse monoclonal antibody (CD147, Thermo Fisher) at room temperature for 30 min in a moisture chamber. Binding of the primary antibody was detected using a dextran-labeled polymer (Dako) for 20 min. Visualization was achieved by adding 3,3′-diaminobenzidine (DAB) as a chromogen, resulting in the characteristic brown stain marking the antibody-enzyme complex. The sections were counterstained with Mayer's hematoxylin for one minute, washed in running tap water for 7–10 min (bluing), dehydrated, cleared, and mounted according to standard procedures [31].

## 3. Results

### 3.1. Statistical Analysis

Statistical analysis was performed using SPSS 16. Data were presented as frequencies and percentages, and an association analysis was conducted using the Chi-square test with a significance threshold of *p* < 0.05.

**Figure 1:**
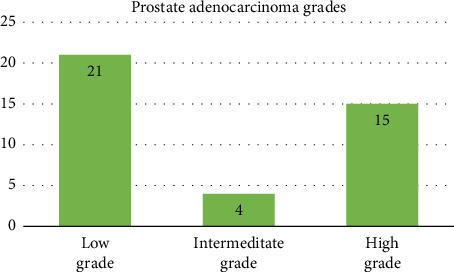
The different grades of adenocarcinoma of the prostate.

The patients' ages ranged between 50 and 79 years, with a mean age of 66.6 years, while the control group ranged from 50 to 82 years, with a mean age of 61.9 years (Table 1). Tumor grade distribution in adenocarcinoma cases revealed that 21 (52.5%) were low grade, 4 (10%) were intermediate grade, and 15 (37.5%) were high grade (Figure 1).

### 3.2. Evaluation of Staining

**Figure 2:**
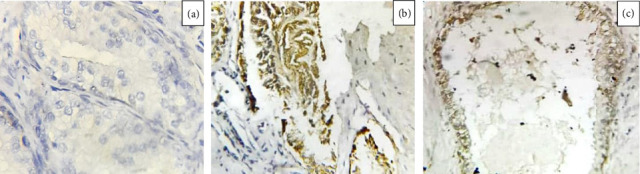
(a) Prostate adenocarcinoma (40x) immunohistochemically staining negative for CD147. (b) Prostate adenocarcinoma (40x) immunohistochemically staining positive for CD147. (c) Prostatic hyperplasia (40x) positive for immunohistochemically staining for CD147.

The staining assessment involved initially scanning the entire tissue specimen at low magnification (×40), followed by detailed confirmation at higher magnifications (×200 and ×400). Slides displaying immunoreactivity were scored as positive, while those without any immunoreactivity were scored as negative (Figure 2). We used this method to generally assess the expression levels.

The expression of CD147 was observed in 9 (22.5%) of the cases and 3 (7%) of the controls (Figure 2). Statistical analysis showed a *p* value of 0.07, an odds ratio of 3.6, and a 95% confidence interval (CI) of 0.9–14.4 (Table 1). Although CD147 expression was higher in cases compared to controls, the association did not reach statistical significance (Table 2).

## 4. Discussion

CD147, also known as basigin or EMMPRIN, has been extensively studied for its role in promoting tumor aggressiveness through the activation of MMPs and modification of the tumor microenvironment [32]. CD147 is highly concentrated in malignant tumors [33]; it can enhance tumor invasion and metastasis by increasing MMP expression [34]. In addition, it is vital in protecting tumor cells that are involved in glycolysis [35].

This study identified differences in CD147 expression between the two groups; however, the statistical analysis found no association between CD147 expression and prostate adenocarcinoma. This outcome contrasts with prior studies that have proposed CD147 as a marker linked to tumor progression and metastasis [23, 30]. The nonsignificant *p* value suggests caution in interpreting these findings, as the lack of statistical significance may be due to the relatively small sample size, which could limit the study's power to detect a meaningful association [36].

The study revealed that low-grade tumors were predominant, accounting for 21 cases in this cohort. This finding aligns with the existing literature, which indicates that lower grade tumors are more commonly observed in early-stage diagnoses. [37]. Interestingly, while CD147 has been linked to higher tumor grades and poorer prognoses in several studies, our findings did not show a clear association between CD147 expression and tumor grade. Although some studies showed mixed results, indicating variability in CD147 expression between different grades of prostate cancer depending on population studied [29, 38–42], some studies have reported that CD147 overexpression correlates with advanced tumor stages and higher grades, contributing to invasive behavior through increased MMP activity [43]. The future research should include larger, multicenter cohorts with diverse populations and well-stratified tumor grades, alongside advanced molecular techniques such as RT-qPCR, western blot, and proteomics to precisely quantify CD147 expression.

## 5. Conclusion

This study highlights the complexity of CD147 expression in prostate adenocarcinoma, with findings showing no statistically significant association between CD147 expression and tumor presence or grade. While CD147 expression was higher in adenocarcinoma cases than in controls, the lack of significance may be influenced by the limited sample size and predominantly low-grade tumors in the cohort. These results underscore the variability of CD147 expression across different populations and tumor grades, as well as the challenges in its interpretation as a biomarker. The future research should focus on larger, multicenter studies involving diverse populations, employing quantitative and functional methods such as RT-qPCR and proteomics to validate CD147's clinical utility. Exploring the mechanistic role of CD147 in tumor progression through functional assays and preclinical models is also crucial to establish its prognostic and therapeutic potential in prostate cancer.

## Figures and Tables

**Table 1:** Study participant age distribution and tumor grade distribution.

Characteristics	Adenocarcinoma cases	Control group
Age range (years)	50–79	50–82
Mean age (years)	66.6	61.9
Tumor grade distribution		
Low grade	21 (52.5%)	—
Intermediate grade	4 (10%)	—
High grade	15 (37.5%)	—

**Table 2:** The degree of association of the cd147 with adenocarcinoma of the prostate.

	Positive	Negative	*p* value	Odds ratio	95% CI
Cases	9	31	0.07	3.6	0.9–14.4
Controls	3	37			

## Data Availability

The data that support the findings of this study are available from the corresponding author on request. The data are not publicly available due to privacy or ethical restrictions.
